# Performance-based assessments and their correlations with self-reports and informant judgments of cognitive and social cognitive impairments in patients with Schizophrenia and Bipolar Disorder

**DOI:** 10.1192/j.eurpsy.2025.1057

**Published:** 2025-08-26

**Authors:** J. Henry, C. A. Depp, P. D. Harvey, A. E. Pinkham, R. C. Moore

**Affiliations:** 1 University of Miami Miller School of Medicine, Miami; 2 UCSD Medical Center, San Diego; 3 UT Dallas, Dallas, United States

## Abstract

**Introduction:**

Cognitive impairment in serious mental illness (SMI) is a major determinant of disability across employment, independence, and social outcomes. However, performance-based assessments are commonly unavailable in clinical settings. The remaining choices are self-report vs. clinician impressions and it is not clear across SMI how much clinician vs self-reports are related to objective performance.

**Objectives:**

In this study, we examine performance-based assessments and their correlations with self-reports and informant judgments of cognitive and social cognitive impairments.

**Methods:**

Participants with bipolar disorder (BPl; n=114) and schizophrenia (SCZ; n=126) participated in a comprehensive study of cognition and functioning, including clinical (MADRS), performance-based assessments, self-reports, and observer ratings of cognitive and social cognitive functioning following a 30-day Ecological Momentary Assessment (EMA) assessment period daily assessments of Positive (PA) and Negative (NA) Affect were collected.

**Results:**

In participants with schizophrenia, self-reports of cognitive functioning were uncorrelated with objective data (r=-.02) and with clinician ratings assessments(r-.00), but were correlated with clinician ratings of depression (r=.41) and EMA reports of momentary NA (r=.37). Interestingly, clinician ratings of cognitive performance were correlated with performance data (r= .41) and MADRS (r=.35) but not self-reported cognitive ability (r=-.08). In participants with bipolar disorder, self-reports of performance ability was correlated with performance-based data (r=.43) and clinicians’ ratings of cognitive ability (r=.51). Both MADRS ratings and EMA-based momentary NA ratings were also correlated with self-reported cognitive performance (r>.44), but not objective cognitive performance (r=.14).

**Conclusions:**

There are clear differences in the ability to self-report cognitive performance in schizophrenia and bipolar disorder. However, clinicians appear to be equivalently able to index cognitive impairment across BPI and SCZ. Participants with BPI appear to be much more able to identify their impairments than those with SCZ, but the correlation between clinician ratings and objective performance seems similar across conditions. These data suggest that reliance on self-reports of impairment may require adjustments based on diagnosis or other factors, but that clinician impressions of cognitive impairment converge with cognitive data.

**Disclosure of Interest:**

None Declared

